# Dynamic pathways to academic engagement in university students: a mixed-methods study on growth mindset, grit, and academic self-efficacy with ecological momentary assessment

**DOI:** 10.3389/fpsyg.2025.1653578

**Published:** 2025-10-22

**Authors:** Haizhen Liang, Shimin Wu

**Affiliations:** ^1^School of Literature and Media Communication, Lingnan Normal University, Zhanjiang, Guangdong, China; ^2^Department of Mathematics, Zhanjiang Preschool Education College, Zhanjiang, Guangdong, China

**Keywords:** growth mindset, academic grit, academic self-efficacy, academic engagement, mediation, mixed-methods, ecological momentary assessment (EMA), real-time dynamics

## Abstract

**Introduction:**

This mixed-methods study investigated the predictive influence of growth mindset, academic grit, and academic self-efficacy on the academic engagement of undergraduate students in mainland China. A key ancillary goal was to explore the real-time dynamics and psychological mechanisms underpinning these relationships.

**Methods:**

We surveyed 593 students from three diverse Chinese universities to assess the core constructs. The quantitative data were analyzed using Structural Equation Modeling (SEM). To provide qualitative depth, we conducted focus group interviews (*n* = 3 groups) and collected Ecological Momentary Assessment (EMA) diaries from a subset of 30 participants over 14 days.

**Results:**

The SEM analysis showed that both growth mindset and academic grit positively predicted academic self-efficacy and engagement. Importantly, academic self-efficacy was found to partially mediate these relationships. Qualitative findings corroborated the model, revealing three key themes: (a) embracing challenges as learning opportunities, (b) self-efficacy as a cornerstone for proactive engagement, and (c) the role of cultural factors in shaping motivation. The EMA diaries provided granular, *in-situ* insights, illuminating how momentary perceptions of competence directly trigger or inhibit active academic involvement.

**Discussion:**

This study underscores the importance of fostering growth mindset, grit, and especially self-efficacy to enhance academic engagement among Chinese undergraduates. The findings highlight the value of a mixed-methods approach in developing a dynamic, culturally-grounded understanding of student motivation and point toward implications for creating culturally sensitive and moment-aware educational interventions.

## 1 Introduction

Fostering robust academic engagement among undergraduates is paramount in higher education, particularly within competitive academic environments like China, where educational attainment is crucial for societal and individual progress. Understanding its drivers is thus nationally significant. Academic engagement, a multifaceted construct encompassing behavioral, emotional, and cognitive dimensions, predicts positive learning outcomes (Appleton et al., 2008; Fredricks et al., 2004). Among factors influencing student success, motivational constructs such as growth mindset (the belief that abilities develop through effort; Dweck, 2006), academic grit (perseverance and passion for long-term goals; Duckworth et al., 2007), and academic self-efficacy (students' confidence in their academic capabilities; Bandura, 1997) are especially salient. In particular, academic self-efficacy is known to directly influence students' willingness to approach challenges and persist in learning activities (Pajares, 1996; Schunk, 1991).

While the contributions of these constructs are well-documented in Western, individualistic contexts, their theoretical application to a non-Western setting requires careful consideration. Specifically, the Chinese cultural landscape, shaped by Confucian heritage, is largely defined by collectivism. This worldview fosters an interdependent self-construal, where an individual's identity, motivations, and sense of accomplishment are deeply intertwined with their role in social units like the family and community (Markus and Kitayama, 1991; Oyserman et al., 2002). This contrasts sharply with the independent self-construal common in individualistic cultures, which prioritizes personal attributes and individual achievement. It is important to note that contemporary Chinese youth often navigate a complex identity, balancing these traditional collectivist expectations with the individualistic pressures of a globalized, market-oriented society (Yan, 2020; Yang, 2018). This unique cultural-psychological tension forms a critical backdrop for understanding student motivation.

This theoretical framework suggests that the mechanisms of Western-derived motivational constructs may manifest differently in Chinese students. For instance, a growth mindset may resonate strongly with the ingrained cultural belief that effort and diligence are virtues that lead to mastery and success. This aligns with the Confucian emphasis on self-perfection through disciplined effort and the widely held belief that academic success is attainable through hard work rather than innate talent (Hau and Salili, 1991; Li, 2009, 2012). The common adage “吃苦” (*chī kǔ*), or enduring hardship, further frames perseverance not just as a strategy but as a moral good. Similarly, academic grit may be fueled not only by personal passion but also by a powerful sense of duty and long-term obligation to one's family, transforming individual perseverance into a collective endeavor. This notion of socially oriented achievement motivation, where personal goals are intertwined with familial and societal expectations, is a well-documented feature of Confucian-heritage cultures (Rozman, 2014; Salili, 1996; Yu and Yang, 1994). Academic self-efficacy, in turn, might be conceptualized less as confidence in one's isolated abilities and more as confidence in one's capacity to meet social and familial expectations (Klassen, 2004).

To articulate the expected interrelations among our variables, we propose a conceptual model grounded in Bandura's (1997) social cognitive theory. In this framework, growth mindset and academic grit act as influential personal factors that build academic self-efficacy, which in turn functions as the direct psychological mechanism driving academic engagement. Specifically, a growth mindset is theorized to boost self-efficacy by reframing academic challenges as learning opportunities rather than threats to innate ability (Dweck, 2006; Yeager and Dweck, 2012). Academic grit is thought to build self-efficacy through perseverance; by persisting through difficulty, students accumulate “mastery experiences”—successful outcomes that are the most powerful source of academic confidence (Fathi et al., 2024; Wolters and Hussain, 2015). Thus, self-efficacy is not just an outcome but a pivotal mediator: the task-specific confidence that translates broader beliefs and dispositions into the focused effort required for academic engagement (Pajares, 1996). This model provides the conceptual basis for our hypotheses.

While the cultural sensitivity of engagement is widely recognized (Santos et al., 2023), scholars have specifically highlighted the need for more research on how these core motivational constructs operate within the unique, high-stakes educational systems of East Asia, where Western models may not fully apply (e.g., King and McInerney, 2014; Li, 2012). Deeper investigation into the mediating pathways in this context is therefore crucial for building a culturally-grounded model of academic engagement. Furthermore, much existing research relies on retrospective self-report, failing to capture the dynamic, fluctuating nature of daily motivational beliefs and engagement.

Given this theoretical backdrop, this study comprehensively explores the relationships among growth mindset, academic grit, academic self-efficacy, and academic engagement in Chinese undergraduate students. Employing a sophisticated mixed-methods design (Creswell and Plano Clark, 2018), we integrate quantitative surveys for statistical relationships, qualitative focus group interviews for rich contextual insights, and innovative Ecological Momentary Assessment (EMA) diaries for real-time dynamics. This approach aims to quantify direct and indirect effects, unpack culturally specific nuances shaping their manifestation and impact on academic engagement, and understand moment-by-moment processes. Ultimately, this study contributes to a culturally informed and practically relevant understanding of student motivation, offering insights for targeted interventions to cultivate engaged and resilient learners in China and similar educational settings.

## 2 Review of prior research

### 2.1 Student engagement: a multifaceted construct

Student engagement, a complex and multifaceted construct, integrates the behavioral, emotional, and cognitive dimensions of student involvement in learning (Fredricks et al., 2004). Recognized as central to educational psychology (Appleton et al., 2008), this key construct captures students' focus, inquisitiveness, and dedication, serving as a critical indicator of academic success, persistence, and overall wellbeing.

The conceptualization of student engagement has evolved. Initially defined by behavioral (e.g., effort, participation), emotional (affective responses), and cognitive (investment in learning) aspects (Fredricks et al., 2004; Skinner and Pitzer, 2012), it later expanded to include academic engagement, emphasizing task persistence (Appleton et al., 2008), and agentic engagement, highlighting students' proactive role in shaping their learning (Reeve and Tseng, 2011). This dynamic process is continuously shaped by contextual factors like instructional quality and peer interactions (Reschly and Christenson, 2012; Wang and Holcombe, 2010). Longitudinal studies demonstrate engagement's significant mediating role between school climate and student outcomes, particularly for underserved populations (Rumberger and Rotermund, 2012), underscoring its systemic nature within ecological frameworks (Bronfenbrenner and Morris, 2006; Järvelä et al., 2016).

Measurement methods for student engagement have advanced from self-report surveys (Appleton et al., 2006; Fredricks et al., 2019) to multi-method approaches. Experience sampling captures real-time shifts in engagement (Shernoff et al., 2016), while neurophysiological tools like eye-tracking and EEG offer objective physiological indicators (Henrie et al., 2015). Despite these advancements, challenges persist in accurately measuring engagement, with critics noting the potential for superficial participation to be mistaken for deep involvement (Sinatra et al., 2015).

Student engagement results from a complex interplay of individual, emotional, and social factors, with significant consequences for learning. Individual differences create a spectrum of engagement profiles, reflecting diverse needs (Lawson and Masyn, 2015). Emotions are critical; positive emotions like enjoyment enhance engagement, while negative ones like anxiety or boredom diminish it (Garn et al., 2017; Pekrun and Linnenbrink-Garcia, 2012). Social-emotional competencies and emotional intelligence also bolster engagement by fostering resilience and positive emotional connections to learning, although effects may vary by context and culture (Maguire et al., 2017; Santos et al., 2023; Thomas and Allen, 2021). These factors collectively impact academic achievement and wellbeing, emphasizing the need for targeted support.

The educational setting profoundly influences student engagement. Classroom practices promoting student autonomy, culturally responsive teaching, and mastery-oriented goals effectively foster engagement (Howard, 2013; Linnenbrink-Garcia et al., 2011; Nasir et al., 2021; Ryan and Deci, 2017). Conversely, punitive discipline and stereotype threat can erode it (Gregory et al., 2010; Nasir et al., 2021). Strong teacher-student relationships are particularly vital for at-risk students (Quin, 2017; Roorda et al., 2011). In digital learning, gamification and collaborative activities enhance engagement, though unequal technology access can exacerbate disparities (Henrie et al., 2015; Sailer and Homner, 2020). The COVID-19 pandemic highlighted challenges in maintaining engagement remotely due to reduced social interaction (Zaccoletti et al., 2020). Critiques of traditional models also point to their oversight of structural inequities and broader sociocultural factors, advocating for more inclusive, contextually aware approaches (Bingham and Okagaki, 2012; Coll et al., 2012; Nasir et al., 2021).

### 2.2 Growth mindset and academic engagement

Growth mindset refers to the belief that one's abilities and intelligence can be developed through dedication and effort (Dweck, 2006). Research consistently shows that students who hold this belief demonstrate greater academic engagement, manifesting as increased effort, persistence, and active learning compared to those with a fixed mindset (Grant and Dweck, 2003; Hong et al., 1999; Vestad and Bru, 2024). They also report increased enjoyment, curiosity, and a sense of belonging (Boaler, 2016; Li, 2023), and are more likely to embrace challenges, use effective learning strategies, and exhibit metacognitive awareness (Blackwell et al., 2007; Mangels et al., 2006). These positive effects appear across diverse educational settings and populations, from foreign language acquisition (Derakhshan et al., 2024; Li, 2023) and early childhood—where it enhances both engagement and wellbeing (Lam et al., 2023)—to university students during the COVID-19 pandemic (Zhao et al., 2021). Interventions promoting intelligence malleability further enhance engagement by boosting motivation and resilience (Aronson et al., 2002; Good et al., 2003; Haimovitz and Dweck, 2016).

The link between growth mindset and academic engagement is often mediated by psychological factors. For example, enjoyment can act as an intermediary, as growth-minded students find learning more intrinsically rewarding, increasing their engagement (Li, 2023). Similarly, in foreign language classrooms, a growth mindset can reduce boredom, sustaining interest (Derakhshan et al., 2024; Zhang et al., 2024). During the COVID-19 pandemic, lower perceived stress contributed to maintaining engagement, enabling growth-minded students to stay involved despite external pressures (Zhao et al., 2021). These examples show how emotional and cognitive elements translate a growth mindset into active learning. However, the strength of this relationship also varies with context. Teacher support can significantly amplify a growth mindset's positive effects, especially where student motivation often wanes (Vestad and Bru, 2024), and a supportive classroom environment can reduce negative emotions like boredom (Derakhshan et al., 2024). Conversely, external stressors, such as perceived crisis intensity during the pandemic, may weaken this association (Zhao et al., 2021). These findings highlight the crucial role of the surrounding environment in fostering student engagement.

Despite the generally positive findings linking a growth mindset to beneficial academic behaviors, some methodological concerns and conceptual nuances have been raised. A meta-analysis by Macnamara and Burgoyne (2023) found growth mindset interventions had limited impact on academic achievement, with small effect sizes that diminished when accounting for publication bias and study quality. This meta-analysis raised concerns about study design, reporting, and potential researcher bias. While focusing on achievement, it prompts questions about generalizing mindset effects to engagement, underscoring the need for more rigorous research to establish causality. Adding to this complexity, recent research challenges the simple binary of growth versus fixed mindsets by conceptualizing them as part of a broader system of beliefs. For instance, Chaffee and Noels (2022) used a person-centered approach to identify mindset profiles among language learners. While they identified distinct growth (20.5%) and fixed (21.8%) profiles, they found the majority of students (57.7%) endorsed a “mixed” profile with varied patterns of goals, anxiety, and persistence. Crucially, their findings suggest that mindsets function as part of a system; although the growth profile was associated with the highest engagement and grades, mindset alone was not a direct predictor of achievement. This highlights that mindsets interact with related factors to influence outcomes, offering a more nuanced understanding of their role in engagement.

### 2.3 Grit and academic engagement

Academic grit, as sustained perseverance and passion for long-term goals (Duckworth et al., 2007), significantly predicts student engagement. This personality trait enhances engagement by fostering resilience and sustaining motivation in learning (Hodge et al., 2018; Wolters and Hussain, 2015).

Research consistently shows a positive association between grit and student engagement across diverse settings. Grittier students exhibit greater effort and persistence in their academic work (Hodge et al., 2018; Wolters and Hussain, 2015), actively remaining involved even in demanding situations. For example, grit predicts engagement and overall academic success in university students (Hodge et al., 2018) and links to self-regulated learning strategies, indicating both cognitive and behavioral engagement (Wolters and Hussain, 2015). This suggests grit drives active participation and resilience in various learning models, from traditional to flipped classrooms (Yoon et al., 2020) and blended learning (Gao et al., 2024).

The relationship between grit and engagement is often mediated by psychological factors that vary by context. Key mediators include “negotiable fate”—the belief in one's ability to influence circumstances (Yau and Shu, 2023)—and online learning self-efficacy (Derakhshan and Fathi, 2024). In blended learning for ESL students, L2 grit and intended effort mediate teacher support's influence on engagement (Gao et al., 2024). Furthermore, in flipped classrooms, engagement itself can mediate, linking grit to perceived academic achievement (Yoon et al., 2020). These studies indicate grit's complex influence on engagement, often depending on specific learning environment processes.

Grit comprises two components: perseverance of effort and consistency of interest. Evidence suggests perseverance of effort is a more reliable predictor of engagement, consistently correlating with self-regulated learning and adaptability (Wolters and Hussain, 2015; Yau and Shu, 2023). Consistency of interest, however, shows weaker, more situation-dependent effects (Yau and Shu, 2023). These findings suggest that sustained effort may be a more crucial factor in fostering engagement than consistent interest. The educational context also profoundly shapes the grit-engagement relationship. For instance, the COVID-19 pandemic highlighted negotiable fate's role in amplifying grit's positive effect (Yau and Shu, 2023). Online and blended learning emphasize self-efficacy and intended effort as mediators (Derakhshan and Fathi, 2024; Gao et al., 2024). In flipped classrooms, professor support for student autonomy interacts with grit to enhance both engagement and perceived achievement (Yoon et al., 2020). These findings suggest that the strength and nature of this relationship depend on the learning environment, implying a need for context-specific grit and engagement interventions.

### 2.4 Self-efficacy in academic contexts

Academic self-efficacy, a core concept in Bandura's social cognitive theory (Bandura, 1977, 1986, 1997), refers to a student's belief in their capability to successfully manage and complete academic tasks (Bandura, 1986; Pajares, 1996). By fostering confidence, this belief crucially shapes student engagement, motivating students to exert effort, persist, and employ effective learning strategies.

Extensive research consistently links academic self-efficacy to greater student engagement. Students with higher self-efficacy demonstrate increased effort and persistence (Pintrich and De Groot, 1990; Schunk and Zimmerman, 1998). A meta-analysis confirmed self-efficacy as a strong predictor of behavioral engagement, with varying effect strengths by educational level (Chang and Chien, 2015). This connection extends to emotional engagement (more enjoyment, less anxiety; Cassidy, 2016; Pekrun et al., 2002) and cognitive engagement (use of advanced strategies like critical thinking; Zimmerman and Martinez-Pons, 1990). Self-efficacy often amplifies motivation, driving both engagement and academic success (Bandura, 1991; Dogan, 2015), establishing it as fundamental for active student participation.

However, self-efficacy's influence on engagement is dynamic, shaped by context and individual psychology, particularly in diverse learning environments. Teacher support in online learning, for instance, significantly boosts both self-efficacy and engagement (Huang and Wang, 2023). Similarly, a student's sense of belonging enhances self-efficacy and resilience, strengthening all dimensions of engagement in distance education (Yi et al., 2024). Emotional wellbeing also plays a role: loneliness can weaken self-efficacy and digital learning engagement, while humor can mitigate these negative effects (Ramli et al., 2024). Additionally, self-regulated learning environments and positive peer interactions reinforce self-efficacy's impact (Schunk, 1991; Schutz and Pekrun, 2007). These elements collectively suggest that external support and emotional state can either strengthen or weaken the self-efficacy-engagement relationship, especially in non-traditional settings.

Self-efficacy's impact extends to academic performance, often with engagement acting as a crucial intermediary. Students with strong self-efficacy tend to engage more deeply, leading to improved grades and greater persistence (Meng and Zhang, 2023; Robbins et al., 2004). This mediating role of engagement highlights its critical link between self-efficacy and academic success. Conversely, low self-efficacy can trigger disengagement, resulting in reduced effort and poorer outcomes (Bandura, 1986; Eccles and Wigfield, 2002). Longitudinal studies further show early self-efficacy predicts sustained engagement and long-term academic achievement (Linnenbrink-Garcia et al., 2018; Wigfield and Eccles, 2000). These findings underscore the importance of fostering self-efficacy to promote greater engagement and, ultimately, enhance academic results.

### 2.5 The study objectives and hypotheses

This study aims to provide a nuanced, culturally grounded understanding of academic engagement among Chinese undergraduates. While existing research highlights the roles of growth mindset, academic grit, and academic self-efficacy in predicting positive academic outcomes, much of this work originates from Western contexts. Therefore, our primary quantitative objective is to examine these relationships within a Chinese undergraduate population.

Based on established literature, we propose the following hypotheses:

- *Hypothesis 1:* Growth mindset will positively predict academic engagement among Chinese undergraduates. A growth mindset encourages students to view effort as a path to mastery and embrace challenges, thereby increasing behavioral and cognitive engagement (Blackwell et al., 2007; Dweck, 2006; Grant and Dweck, 2003; Mangels et al., 2006; Yeager and Dweck, 2012).
- *Hypothesis 2:* Academic grit will positively predict academic engagement in this cultural context. Grit motivates students to persist through academic obstacles and maintain focus (Duckworth et al., 2007; Duckworth and Quinn, 2009; Eskreis-Winkler et al., 2014; Hodge et al., 2018; Wolters and Hussain, 2015).
- *Hypothesis 3:* Academic self-efficacy will partially mediate the positive relationships between both growth mindset and academic grit with academic engagement among Chinese undergraduates. Rooted in Bandura's social cognitive theory (Bandura, 1977, 1986, 1997), self-efficacy—the belief in one's capability to execute actions for desired attainments—is a critical mechanism translating motivational beliefs into academic behaviors (Pajares, 1996; Schunk and Zimmerman, 1998). Prior research indicates self-efficacy predicts engagement and mediates between broader motivational constructs and outcomes (Chang and Chien, 2015; Dogan, 2015; Huang and Wang, 2023; Li, 2023; Meng and Zhang, 2023; Ramli et al., 2024; Yau and Shu, 2023). Understanding this pathway is crucial for designing targeted interventions.

Beyond these quantitative objectives, a critical purpose of this study is to enrich statistical findings with in-depth qualitative insights into Chinese undergraduates' lived experiences. While quantitative methods establish relationship strength, they often lack nuanced contextual understanding. The qualitative component, utilizing focus group interviews, aims to explore how Chinese undergraduates perceive and experience growth mindset, grit, self-efficacy, and academic engagement in their daily lives. Crucially, Ecological Momentary Assessment (EMA) diaries provide a second, innovative qualitative objective: to capture the *in-situ*, moment-to-moment fluctuations and activation of these motivational beliefs and academic engagement within students' natural academic environments. This provides a dynamic, process-level understanding that complements retrospective insights. This combined approach will yield rich, descriptive data, identifying culturally specific nuances, potential discrepancies between quantitative trends and lived experiences, and a deeper understanding of contextual factors shaping motivation and engagement within the Chinese educational environment. By integrating these diverse quantitative and qualitative approaches, this study seeks to offer a more comprehensive understanding of motivational factors driving academic engagement, ultimately contributing to culturally sensitive and effective educational practices.

## 3 Methods

### 3.1 Participants

The participant group for this study consisted of 593 undergraduate students, purposefully recruited from three distinct and reputable universities across mainland China. To ensure a geographically representative sample, universities were selected from the Northern (Beijing Normal University, a comprehensive research university known for its humanities and social sciences programs), Central (Huazhong University of Science and Technology, a large multi-disciplinary university with a strong emphasis on engineering and business), and Southern Coastal (Shenzhen University, a university specializing in international business and economics) regions of China, thereby capturing potential regional variations in educational environments and student demographics. The final sample comprised 327 female students and 266 male students, a gender distribution closely mirroring the typical undergraduate enrollment ratios in social sciences and business-related disciplines within Chinese higher education. The age of participants ranged from 18 to 24 years (*M* = 20.32, *SD* = 1.87), aligning with the expected age range for full-time undergraduate students in China. The majority of participants were in their second (45%) and third (38%) years of undergraduate study, with smaller proportions in their first (12%) and fourth (5%) years; this distribution was considered advantageous as it focused on students who had navigated the initial transition to university life and were deeply engaged with their chosen academic paths. While the participant pool represented a variety of academic disciplines, a deliberate effort was made to recruit from programs where motivational constructs such as mindset, grit, and self-efficacy are particularly relevant and actively discussed, including psychology (28%), education (25%), business administration and management (32%), and related social science fields (15%).

Participants were recruited via convenience sampling, with study announcements made in large undergraduate courses within the aforementioned departments; instructors kindly facilitated brief recruitment presentations at the beginning or end of regular class sessions. Participation was entirely voluntary, and all students provided informed consent prior to completing the questionnaires; they were assured of anonymity and confidentiality, and no course credit or monetary compensation was offered to minimize potential coercion and ensure intrinsic motivation for participation. The research protocol, encompassing recruitment, informed consent, and data collection procedures, was rigorously reviewed and granted ethical approval by the Lingnan Normal University Research Ethics Committee, affirming the study's adherence to established ethical principles for research involving human subjects. The achieved sample size of 593 was determined a priori to be statistically sufficient for the planned mediation analyses, based on statistical power calculations using G^*^Power and assuming a medium effect size (f^2^ = 0.15), thus ensuring adequate statistical power (above 0.90) to detect hypothesized relationships between the study variables with a reasonable degree of confidence.

### 3.2 Instruments

This study employed a mixed-methods design, utilizing both quantitative and qualitative instruments. The quantitative phase relied on self-administered questionnaires to evaluate growth mindset, academic grit, academic self-efficacy, and academic engagement. Established instruments utilizing Likert-type response formats were employed to ensure robust and reliable measurement of each construct. Prior to the main data collection, we conducted a two-stage process to ensure the face validity and cultural appropriateness of all questionnaire items for Chinese undergraduates (Allen et al., 2023). First, a panel of three bilingual professors in educational psychology at a participating university reviewed all translated and adapted items for conceptual equivalence, cultural relevance, and linguistic clarity. Second, following the expert review, we conducted a pilot study with 30 undergraduate students (who did not participate in the main study) to gather feedback on item comprehensibility and perceived relevance. Feedback from the pilot study was positive, confirming that the items were clear and directly related to students' academic experiences. Based on this process, minor wording adjustments were made to two items on the Academic Grit Questionnaire to enhance clarity before the final survey was distributed. The qualitative phase incorporated semi-structured focus group interviews to explore and contextualize the quantitative findings.

#### 3.2.1 Growth mindset scale

To assess growth mindset, items reflective of Dweck's (2006) conceptual framework on mindset were utilized. This instrument is designed to gauge individuals' beliefs regarding the malleability of their intellectual capabilities and talents. Exemplifying the scale's content are items such as: “Your intelligence is something very basic about you that you can't change very much” (reverse-scored) and “You can always substantially change how intelligent you are.” Participants responded to each item using a Six-point Likert scale, with anchors ranging from 1 (Strongly disagree) to 6 (Strongly agree). A composite score for growth mindset was derived by averaging item scores, where elevated scores are indicative of a stronger endorsement of a growth mindset. We used the Chinese version of the Growth Mindset Scale (Chen et al., 2023), which was developed and validated for use with Chinese student samples. In the current study, the scale demonstrated good internal consistency (α = 0.87).

#### 3.2.2 Academic grit questionnaire

Academic grit, defined as the perseverance and passion for long-term academic objectives, was measured with the Academic Grit Questionnaire (Clark, 2017). This 10-item measure is specifically tailored to the academic domain. Representative items include statements like: “Setbacks don't discourage me in my academic pursuits” and “I am diligent in my studies.” Participants indicated their agreement level with each item using a Five-point Likert scale, ranging from 1 (Strongly disagree) to 5 (Strongly agree). A total grit score was computed by summing item responses, with higher scores signifying greater academic grit. The Academic Grit Questionnaire was translated into Chinese using a standard back-translation procedure to ensure linguistic equivalence. Its internal consistency in this sample was strong (α = 0.86).

#### 3.2.3 Academic self-efficacy scale (ASES)

Academic self-efficacy, referring to students' confidence in their capacity to succeed in academic tasks, was evaluated using the Academic Self-Efficacy Scale (ASES; Pintrich and De Groot, 1990). The ASES, a widely recognized instrument, includes items such as: “I am confident that I can understand the course material taught in this class” and “I am certain I can master the skills being taught in this class.” Responses were recorded on a Five-point Likert scale, ranging from 1 (Strongly disagree) to 5 (Strongly agree). Overall academic self-efficacy scores were calculated by averaging across all items, with higher scores reflecting stronger academic self-efficacy beliefs. We administered a Chinese version of the ASES that has been recently used and confirmed as reliable with Chinese college students (Luo et al., 2023). The scale showed excellent internal consistency in the current study (α = 0.90).

#### 3.2.4 Utrecht work engagement scale for students (UWES-S)

Academic engagement, characterized by vigor, dedication, and absorption in academic pursuits, was measured using the Chinese adaptation (Gan et al., 2007) of the Utrecht Work Engagement Scale for Students (UWES-S; Schaufeli et al., 2002). This 17-item scale assesses engagement across three dimensions: vigor, dedication, and absorption. Illustrative items include: “When I study, I feel like I am bursting with energy” (Vigor), “My studies inspire me” (Dedication), and “I get carried away when I'm studying” (Absorption). Participants rated the frequency of these experiences on a Seven-point Likert scale ranging from 0 (Never) to 6 (Every day). Both a total academic engagement score, as well as subscale scores for vigor, dedication, and absorption, were computed, with higher scores indicating greater academic engagement. The internal consistency for the total UWES-S scale in this sample was high (α = 0.92).

#### 3.2.5 Focus group interviews

To complement the quantitative data and gain deeper qualitative insights, focus group interviews were employed. Three focus group interviews were conducted, each comprising 6–8 undergraduate students purposefully selected from the larger sample to represent a range of academic engagement and self-efficacy levels. The focus groups were semi-structured in nature, guided by an interview protocol that included open-ended questions designed to explore students' perceptions and experiences related to growth mindset, academic grit, academic self-efficacy, and academic engagement within their academic context. The full interview protocol is available in Appendix A. Each focus group session was audio-recorded and subsequently transcribed verbatim to ensure a comprehensive record of the discussions. The qualitative data obtained from these interviews were then analyzed using thematic analysis to identify recurring themes and patterns, providing rich contextual details to support and interpret the quantitative findings.

#### 3.2.6 Ecological momentary assessment (EMA) diaries

To capture real-time, context-dependent experiences of motivation and engagement, a subset of 30 participants from the main sample were recruited to complete structured qualitative diaries using an online form. These participants were purposively selected from the larger survey sample to ensure representation across different levels of academic engagement and self-efficacy, as well as balanced across academic years and universities. Participants were prompted once daily for 2 weeks (14 days) to reflect on and describe specific academic experiences. Daily prompts included:

“Describe a moment today when you felt highly engaged in your studies. What were you doing, and what were you thinking/feeling?”

“Describe a moment today when you faced an academic setback or felt discouraged. How did you react, and what thoughts came to mind?”

“When did your confidence in your academic abilities play a role today? Explain.” These prompts were designed to elicit rich, *in-situ* narratives reflecting the dynamic interplay of growth mindset, academic grit, and self-efficacy in their daily academic lives. Participants submitted their responses via a secure online platform, ensuring data privacy and ease of submission.

### 3.3 Procedures

Data collection occurred during the Fall semester of 2024 (late September to early November), aligning with typical Chinese undergraduate academic workloads. Following ethical approval and institutional permissions, participant recruitment began through announcements in large undergraduate classes. Interested students provided informed consent and completed the questionnaire survey in regular classrooms to ensure a familiar environment. Researchers provided a standardized introduction, reiterated ethical considerations, and distributed questionnaire booklets, presented in a fixed sequence (Growth Mindset Scale, Academic Grit Questionnaire, Academic Self-Efficacy Scale, and UWES-S). Participants completed questionnaires individually (20–25 min), with researchers available for procedural questions. Completed booklets were collected and securely stored.

Subsequently, a qualitative phase—focus group interviews and Ecological Momentary Assessment (EMA) diaries—enriched quantitative findings by exploring student experiences. Focus group participants were purposefully selected from the larger sample based on their Academic Engagement and Academic Self-Efficacy scores, representing a range of engagement levels. Invitations were sent via email, and volunteers attended one of three semi-structured focus groups (6–8 participants each). Interviews, guided by a protocol informed by initial quantitative results and the study's theoretical framework, explored students' understandings and lived experiences of growth mindset, academic grit, self-efficacy, and academic engagement. Each 60–75 min session was audio-recorded for verbatim transcription and later subjected to thematic analysis (Braun and Clarke, 2006).

For the EMA component, a separate subset of 30 participants was recruited after the initial survey, based on their willingness to provide daily reflections. This purposive selection aimed for diverse representation across engagement levels, academic years, and universities. Participants received detailed instructions for submitting daily diary entries via a secure online platform. Reminders were sent daily at a consistent time (e.g., evening) to prompt reflections on the day's academic experiences. EMA data collection spanned two continuous weeks, yielding 14 distinct data points per participant (420 total entries). Researchers monitored submission and provided technical support. A high compliance rate of 96.4% was achieved, with an average of 13.5 entries per participant (405 completed entries). All 29 participants who completed at least 10 entries were included in the EMA qualitative analysis to ensure sufficient data density.

### 3.4 Data analysis

The collected data underwent comprehensive quantitative and qualitative analysis, aligning with the study's mixed-methods design. For quantitative data, initial screening addressed missing values and outliers. Little's MCAR test confirmed data were missing completely at random, allowing expectation-maximization (EM) imputation for the minimal missing points (< 2% of total dataset), thereby preserving statistical power and minimizing bias (Kline, 2016). Descriptive statistics (means, standard deviations, Pearson correlations) provided an initial overview of sample characteristics and inter-relationships among growth mindset, academic grit, academic self-efficacy, and academic engagement. Structural Equation Modeling (SEM), using AMOS software (Byrne, 2010), tested the hypothesized mediation model. This model specified growth mindset and academic grit as exogenous predictors, academic self-efficacy as the mediator, and academic engagement as the primary outcome. Model fit was assessed using CFI, TLI, RMSEA, and SRMR indices, with acceptable fit at CFI/TLI > 0.90, RMSEA < 0.08, and SRMR < 0.08 (Hu and Bentler, 1999). Bootstrapping (5,000 resamples) examined crucial indirect effects, providing robust standard errors and confidence intervals to determine their statistical significance (Preacher and Hayes, 2008).

Complementing the quantitative analysis, qualitative data from focus group interviews and EMA diaries were analyzed using the systematic approach of thematic analysis (Braun and Clarke, 2006). Verbatim transcripts of audio recordings (for focus groups) and daily EMA entries underwent rigorous, iterative familiarization, coding, theme development, and refinement. A hybrid approach of deductive and inductive coding was employed to ensure a comprehensive analysis that was both theory-driven and data-grounded.

The deductive portion of the analysis was guided by a preliminary coding framework designed to systematically identify text related to the study's key constructs. Specifically, coders identified statements reflecting a belief in malleable intelligence as *Growth Mindset* (Dweck, 2006), while expressions of perseverance toward long-term goals were coded as *Academic Grit* (Duckworth et al., 2007). This framework also included coding for *Academic Self-Efficacy*, defined as a student's confidence in their ability to complete academic tasks (Bandura, 1997), and *Academic Engagement*, which encompassed descriptions of behavioral, emotional, or cognitive involvement in learning (Fredricks et al., 2004). At the same time, an inductive analysis was performed to capture important themes that emerged directly from the data and fell outside this predefined framework. This data-driven approach was crucial for identifying nuanced, context-specific factors, revealing, for instance, the pervasive influence of *familial expectations and societal pressure*—a powerful motivator that did not fit neatly into the initial deductive codes.

To ensure coding consistency and trustworthiness across both approaches, two independent researchers initially coded 25% of the qualitative data (transcripts and EMA entries). Inter-coder agreement was high, achieving a Cohen's Kappa of 0.82 for emergent themes, with all discrepancies resolved through discussion and consensus. Following coding, related codes were clustered to form overarching themes, which were then reviewed and refined to ensure they accurately reflected the patterns and meanings within the data. Thematic saturation was determined to be achieved when no new themes or significant properties of existing themes emerged from subsequent data analysis across both focus group and EMA datasets. NVivo software facilitated data management, coding, and theme development. Throughout the qualitative analysis process, researcher reflexivity was maintained through regular debriefing sessions among the research team, where assumptions and interpretations were critically examined to enhance the objectivity and transparency of the findings. The final analysis stage involved interpreting identified themes in relation to quantitative findings, exploring how qualitative data provided richer context and deeper insights into the statistically significant relationships observed in the SEM analysis, thus achieving a more nuanced and integrated understanding of the research question from a mixed-methods perspective (Creswell and Plano Clark, 2018).

## 4 Findings

### 4.1 Quantitative results

The quantitative data analysis was conducted to examine the hypothesized relationships among growth mindset, academic grit, academic self-efficacy, and academic engagement. Preliminary analyses, including descriptive statistics and correlational analyses, provided an initial overview of the data, followed by Structural Equation Modeling (SEM) to test the proposed mediation model.

#### 4.1.1 Descriptive statistics and correlations

Descriptive statistics for all study variables are presented in Table 1, including means, standard deviations, skewness, and kurtosis values. All variables exhibited acceptable ranges and distributions, with skewness and kurtosis values falling within the acceptable limits (±1.0), indicating approximate normality of the data, which is a prerequisite for SEM analysis (Kline, 2016). Pearson correlation coefficients, also displayed in Table 1, revealed significant positive inter-correlations among all study variables. Specifically, both growth mindset (*r* = 0.42, *p* < 0.001) and academic grit (*r* = 0.38, *p* < 0.001) showed moderate positive correlations with academic self-efficacy. Furthermore, growth mindset (*r* = 0.35, *p* < 0.001), academic grit (*r* = 0.41, *p* < 0.001), and academic self-efficacy (*r* = 0.51, *p* < 0.001) all demonstrated moderate to strong positive correlations with academic engagement, suggesting a positive interplay among these motivational and engagement constructs within the academic context of the studied sample.

**Table 1:** Descriptive statistics and inter-correlations of study variables (*N* = 593).

**Variable**	***M***	***SD***	**Skewness**	**Kurtosis**	**1**	**2**	**3**	**4**
1. Growth mindset	4.72	0.85	−0.32	0.15	–			
2. Academic grit	3.91	0.63	−0.18	0.22	0.28^**^	–		
3. Academic self-efficacy	3.78	0.71	−0.25	0.08	0.42^**^	0.38^**^	–	
4. Academic engagement	4.25	0.92	−0.45	0.30	0.35^**^	0.41^**^	0.51^**^	–

^**^p < 0.001.

#### 4.1.2 Testing the hypothesized model

##### 4.1.2.1 Measurement model analysis

Prior to testing the structural relationships, a confirmatory factor analysis (CFA) was conducted to assess the measurement model fit and validate the latent constructs (growth mindset, academic grit, academic self-efficacy, and academic engagement). The measurement model demonstrated good fit to the data [χ^2^(df = 129) = 385.20, *p* < 0.001], with acceptable fit indices: CFI = 0.94, TLI = 0.93, RMSEA = 0.05 (90% CI [0.04, 0.06]), and SRMR = 0.04. Standardized factor loadings for all items ranged from 0.65 to 0.89, indicating good convergent validity. Furthermore, Composite Reliability (CR) values for all constructs ranged from 0.81 to 0.91, exceeding the threshold of 0.70. Average Variance Extracted (AVE) values ranged from 0.55 to 0.75, surpassing the recommended 0.50, and were greater than the squared inter-construct correlations, confirming adequate discriminant validity among the latent variables.

##### 4.1.2.2 Structural model analysis

The hypothesized mediation model, depicting growth mindset and academic grit as predictors of academic engagement mediated by academic self-efficacy, was tested using SEM. The model demonstrated good fit to the data, as indicated by the following fit indices: $\chi_{\left(132\right)}^{2}$ = 401.55, *p* < 0.001, CFI = 0.96, TLI = 0.95, RMSEA = 0.058 [90% CI:0.049, 0.067], SRMR = 0.035. These values meet or exceed commonly accepted criteria for good model fit (Hu and Bentler, 1999), suggesting that the hypothesized model adequately represents the relationships among the variables in the sample.

**Figure 1:**
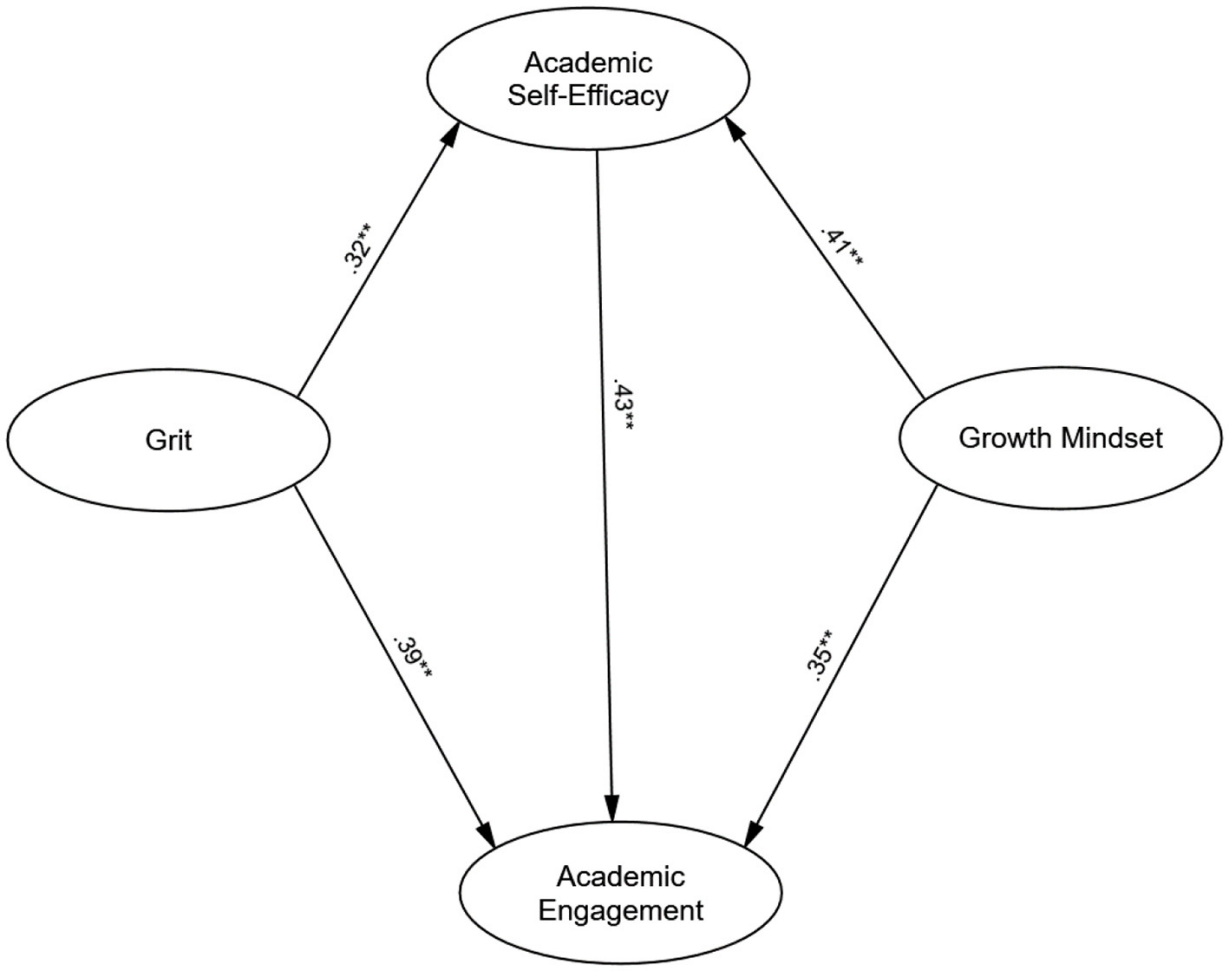
Path diagram of the mediation model with standardized path coefficients. **p < 0.001.

Examination of the path coefficients revealed significant positive direct effects of both growth mindset and academic grit on academic self-efficacy. Specifically, growth mindset significantly predicted academic self-efficacy (β = 0.41, *p* < 0.001), as did academic grit (β = 0.32, *p* < 0.001). Furthermore, academic self-efficacy exhibited a strong positive direct effect on academic engagement (β = 0.43, *p* < 0.001). The direct effects of growth mindset (β = 0.35, *p* < 0.001) and academic grit (β = 0.39, *p* < 0.001) on academic engagement also remained statistically significant. All path coefficients and their statistical significance are summarized in Table 2 and Figure 1.

**Table 2:** Standardized direct, indirect, and total effects (*N* = 593).

**Effect**	**Type**	**β**	***SE***	***p***	**95% CI**
Growth mindset → academic self-efficacy	Direct	0.41	0.09	< 0.001^***^	[0.23, 0.59]
Academic grit → academic self-efficacy	Direct	0.32	0.08	< 0.001^***^	[0.16, 0.48]
Academic self-efficacy → academic engagement	Direct	0.43	0.09	< 0.001^***^	[0.25, 0.61]
Growth mindset → academic engagement	Direct	0.35	0.07	< 0.001^***^	[0.21, 0.49]
Academic grit → academic engagement	Direct	0.39	0.08	< 0.001^***^	[0.23, 0.55]
Growth mindset → academic self-efficacy → academic engagement	Indirect	0.18	0.03	< 0.001^***^	[0.12, 0.25]
Academic grit → academic self-efficacy → academic engagement	Indirect	0.14	0.03	< 0.001^***^	[0.08, 0.20]
Growth mindset → academic engagement	Total	0.53	0.10	< 0.001^*^	[0.33, 0.73]
Academic grit → academic engagement	Total	0.53	0.10	< 0.001^*^	[0.33, 0.73]

^***^p < 0.001.

To formally test the hypothesized mediation effects, bootstrapping procedures with 5,000 resamples were conducted. The results indicated significant indirect effects of both growth mindset and academic grit on academic engagement through academic self-efficacy. Specifically, the indirect effect of growth mindset on academic engagement via academic self-efficacy was significant (β = 0.18, 95% CI [0.12, 0.25], *p* < 0.001), as was the indirect effect of academic grit on academic engagement through academic self-efficacy (β = 0.14, 95% CI [0.08, 0.20], *p* < 0.001). The 95% confidence intervals for both indirect effects did not include zero, providing further support for the presence of significant mediation.

Considering both the significant direct effects of growth mindset and academic grit on academic engagement and the significant indirect effects through academic self-efficacy, the findings suggest partial mediation. Academic self-efficacy partially mediates the relationships between both growth mindset and academic grit with academic engagement, indicating that while growth mindset and grit have direct influences on engagement, a significant portion of their effect is channeled through students' beliefs in their academic capabilities.

The model explained a substantial portion of the variance in academic self-efficacy (*R*^2^ = 0.28) and academic engagement (*R*^2^ = 0.35). Specifically, growth mindset and academic grit, in combination, accounted for 28% of the variance in academic self-efficacy. Furthermore, growth mindset, academic grit, and academic self-efficacy together explained 35% of the variance in academic engagement, highlighting the collective importance of these motivational factors in predicting students' active and involved learning experiences.

### 4.2 Qualitative results

The qualitative phase, utilizing thematic analysis of focus group interviews and Ecological Momentary Assessment (EMA) diaries, provided rich contextual insights that both corroborated and expanded upon the quantitative findings. Three overarching themes emerged from the focus group data, offering a nuanced understanding of how growth mindset, academic grit, and academic self-efficacy intertwine to shape academic engagement among undergraduate students in mainland China: (a) Embracing Challenges and Setbacks as Learning Opportunities, (b) Self-Efficacy as the Cornerstone of Proactive Engagement, and (c) Cultural and Contextual Influences on Motivational Beliefs and Engagement. The EMA data further enriched these themes by providing real-time, *in-situ* accounts of these processes. Each theme is detailed below with illustrative quotes from focus groups and selected EMA entries to provide a vivid and authentic representation of the student experience.

#### 4.2.1 Embracing challenges and setbacks as learning opportunities: the manifestation of growth mindset and grit

This theme captures how students expressed and enacted growth mindset and academic grit. Participants often described viewing academic challenges as valuable opportunities for learning and growth, reflecting growth mindset principles. Lin, from Beijing Normal University, shared: “When I encounter a difficult problem, like in advanced mathematics, my first reaction is not frustration, but curiosity. It's like a puzzle. I think, ‘Okay, this is tough, but if I can solve this, I will really learn something new and become stronger in this subject.'“ She added, “It's not about being naturally smart, but about training my brain,” emphasizing the malleability of cognitive abilities.

Students consistently highlighted persistence and resilience during setbacks, aligning with academic grit. Chen, from Shenzhen University, recounted: “There are times when I feel completely overwhelmed by coursework… But I tell myself that giving up is not an option… I remind myself of my long-term goals… and break down the tasks into smaller, manageable steps. It's about pushing through even when it's uncomfortable.” Wang, also from Shenzhen University, further underscored this proactive approach: “Grit is like fuel. Even when I feel tired or discouraged, I know I need to keep going to reach my destination.”

Furthermore, participants reframed failure as feedback, a key aspect of both constructs. Mei, from Huazhong University of Science and Technology, observed: “If I don't do well on an exam… I don't see it as a reflection of my inherent inability. Instead, I analyze what went wrong… I identify areas where I need to improve, and adjust my study strategies for the next time. It's a continuous process of learning from mistakes.” She continued, “It's like each mistake is a pointer, guiding me to where I need to focus more,” illustrating a proactive and improvement-oriented response. These narratives vividly demonstrate how students with stronger growth mindsets and higher grit levels actively interpret and respond to academic challenges, fostering learning and sustained effort, consistent with the positive correlations found in the quantitative data.

#### 4.2.2 Self-efficacy as the cornerstone of proactive engagement: confidence fuels action

The second theme highlighted academic self-efficacy's pivotal role in driving proactive academic engagement. Students frequently emphasized that confidence in their academic abilities catalyzed their active participation and investment. Zhang, from Beijing Normal University, articulated this: “When I feel confident that I can master the material… I am much more likely to actively participate in class discussions, ask questions, and seek out extra resources… It's like the confidence itself propels me forward.” He further explained, “If I am unsure, I tend to stay quiet and just listen. But when I feel sure, I want to engage more, to test my understanding and contribute to the discussion.”

Li, from Shenzhen University, elaborated on this sense of agency: “If I believe I can succeed in a course… I am more willing to put in the necessary effort, even if it's demanding. I'll spend more time studying, I'll try different learning techniques, and I won't be easily discouraged by difficulties because I believe my efforts will pay off.” She added, “It's like a positive cycle. The more effort I put in, the better I understand, and the more confident I become, which in turn motivates me to engage even more actively.” Focus groups also revealed self-efficacy as not merely about feeling confident, but actively seeking competence enhancement. Sun, from Huazhong University of Science and Technology, stated: “Sometimes I intentionally choose more challenging courses or projects… I know it will be difficult, but I also know that overcoming these challenges will boost my skills and my confidence in the long run. It's a cycle—confidence drives engagement, and engagement builds more confidence.” He concluded, “It's about pushing my own limits and seeing what I am capable of. It's not just about getting a good grade, but about proving to myself that I can learn and grow.” These accounts strongly resonate with the quantitative findings, particularly the robust positive correlation and mediating role of academic self-efficacy, illustrating it as a crucial psychological mechanism translating motivational beliefs into tangible academic actions and engagement.

#### 4.2.3 Cultural and contextual influences on motivational beliefs and engagement

The final theme underscored the significant influence of Chinese cultural and contextual factors on students' motivational beliefs and academic engagement. Participants frequently cited the highly competitive academic environment and societal emphasis on achievement. Zhao, from Shenzhen University, commented: “In China, academic success is so highly valued… there is immense pressure to perform well. This pressure can be stressful, but it also pushes us to work harder and strive for excellence. It's almost like grit is not just a personal trait, but also a cultural expectation.” Liu, from Beijing Normal University, reinforced this: “From a young age, we are taught the importance of hard work and perseverance. ‘No pains, no gains' is a common saying. This cultural emphasis on effort definitely shapes how we approach our studies.”

Focus groups also revealed nuanced perspectives on growth mindset. While generally endorsing malleable intelligence, some students expressed concerns about excessive pressure and self-blame if continuous effort didn't yield immediate success. Fang, from Beijing Normal University, noted: “The growth mindset is encouraging, but sometimes it feels like there's too much emphasis on ‘you can achieve anything if you just try hard enough.' In reality, there are external factors and limitations… It's important to have a growth mindset, but also to be realistic and kind to yourself when things are truly challenging.” She reflected on potential downsides: “It's about finding a balance between pushing yourself and accepting that sometimes things are beyond your control. Otherwise, it can be exhausting and even lead to burnout.” Interestingly, traditional Chinese values like collectivism and filial piety also influenced motivation. Kang, from Huazhong University of Science and Technology, shared: “For many of us, our academic success is not just for ourselves, but also for our families. We feel a strong sense of responsibility to make our parents proud and to contribute to our family's honor. This sense of duty can be a powerful motivator for academic engagement.” He added, “Knowing that my family is sacrificing for my education… makes me want to work even harder and not let them down. It's a way of showing my gratitude and fulfilling my responsibilities as a son.” These insights suggest that while growth mindset, grit, and self-efficacy are universally relevant, their manifestation and impact on academic engagement are shaped by specific cultural and contextual factors within the Chinese higher education system, adding depth to quantitative findings.

#### 4.2.4 Real-time dynamics of motivation and engagement: insights from EMA

The Ecological Momentary Assessment (EMA) diaries provided granular, in-the-moment insights into the fluctuating nature of students' motivation and engagement, offering a dynamic complement to the retrospective accounts from focus groups.

EMA entries frequently captured how students' growth mindset was activated in response to unexpected academic difficulties. For example, a student from Beijing Normal University consistently reported on day 5 of the EMA period: “Felt really stuck on a coding problem for my engineering project. My first thought was, ‘This is hard, but I can figure it out if I keep trying different approaches.' I didn't feel like giving up, just more determined.” This immediate cognitive reframing in the face of a challenge directly exemplifies a growth mindset in action, corroborating the qualitative theme of embracing challenges.

Similarly, EMA entries illuminated the moment-to-moment manifestation of academic grit. A student from Huazhong University of Science and Technology noted on day 9: “Had a huge pile of readings today. Initially felt overwhelmed and procrastinated for an hour. But then remembered my goal for the semester—to deeply understand this subject—and just started with one small paragraph. Managed to finish a significant portion, felt a sense of quiet satisfaction.” This demonstrates perseverance in tackling a tedious task and a sustained connection to long-term academic objectives, even amidst initial inertia.

The EMA data particularly underscored the direct, immediate influence of academic self-efficacy on engagement. Several entries showed a direct correlation between a momentary feeling of competence and increased engagement. A Shenzhen University student's entry on day 12 highlighted: “Just finished a challenging econometrics tutorial problem correctly. Felt really good about my understanding. Immediately wanted to tackle more problems and spent an extra hour practicing. Confidence definitely fueled my desire to engage deeper.” Conversely, another entry on day 7 stated: “Struggled with the statistics lecture today, felt like I couldn't grasp the concepts. Lost motivation quickly and ended up browsing social media instead of reviewing. My confidence just dropped, and so did my focus.” These real-time accounts vividly illustrate how perceived competence directly triggers or inhibits active academic involvement, reinforcing self-efficacy as a cornerstone of proactive engagement.

Furthermore, EMA entries subtly hinted at the cultural and contextual influences, particularly the underlying pressure or sense of responsibility. One student, across several entries, often paired moments of high engagement or perseverance with thoughts of “not letting my parents down” or “contributing to my future.” While not explicitly prompted, these recurring implicit motivations in the immediate context of academic tasks offer a valuable real-time perspective on the cultural factors discussed in the focus groups.

Overall, the EMA findings provided a micro-level, dynamic understanding of the constructs, offering a unique “snapshot” of students' experiences as they unfolded. This real-time data complements the broader narratives from focus groups by showing how motivational beliefs and engagement are activated and sustained (or disrupted) in specific academic moments, further deepening our understanding of their complex interplay within the Chinese educational environment.

In summary, the qualitative findings from focus groups and EMA diaries collectively enrich the quantitative results. The thematic analysis provides compelling firsthand accounts illustrating how growth mindset and academic grit manifest in students' approaches to challenges, how academic self-efficacy facilitates active academic engagement, and how the broader cultural context shapes their motivational landscape. These qualitative insights align with and contextualize the positive relationships identified in the SEM analysis, offering a more holistic and ecologically valid understanding of student motivation and engagement in mainland Chinese universities.

## 5 Discussion

This study investigated the interplay of growth mindset, academic grit, academic self-efficacy, and academic engagement among Chinese undergraduates. Our mixed-methods approach, integrating quantitative surveys with both retrospective focus group interviews and real-time Ecological Momentary Assessment (EMA) diaries, revealed that both growth mindset and academic grit significantly predict academic self-efficacy and academic engagement. Critically, academic self-efficacy partially mediated these relationships. Qualitative data, now enriched by in-the-moment accounts from EMA, further provided nuanced insights into students' lived experiences and highlighted the considerable role of cultural and contextual factors. This discussion interprets these findings, substantiates their importance, explores practical implications, and suggests future research directions.

Regarding the direct influence of motivational beliefs, our quantitative results affirm that growth mindset and academic grit are significant predictors of academic engagement, consistent with prior research. Growth mindset, defined by the belief in malleable abilities, has consistently linked to heightened student engagement, as individuals perceive challenges as opportunities for skill enhancement (Dweck, 2006; Grant and Dweck, 2003; Vestad and Bru, 2024). Similarly, academic grit, characterized by sustained perseverance and passion for long-term goals, catalyzes engagement, motivating students to maintain academic involvement despite obstacles (Duckworth et al., 2007; Hodge et al., 2018; Wolters and Hussain, 2015). Our study's findings mirror those of Hodge et al. (2018), who documented increased effort and participation among gritty students. The robustness of these positive relationships, particularly within China's distinctive cultural and educational milieu—marked by intense academic competition and a strong societal emphasis on achievement—validates the broader relevance and cross-cultural applicability of these motivational constructs. The EMA data further substantiated these direct links by capturing micro-level instances where students' immediate thoughts reflecting growth mindset (e.g., “I can figure this out”) or grit (e.g., “I need to keep going”) directly preceded or accompanied moments of sustained academic effort and engagement.

A key contribution of our study lies in identifying academic self-efficacy's partial mediating role in the relationships between growth mindset, grit, and academic engagement. This finding underscores self-efficacy—the conviction in one's capability to succeed academically—as a crucial psychological conduit translating motivational beliefs into observable academic behaviors. Bandura's social cognitive theory (Bandura, 1977, 1986, 1997) asserts that self-efficacy shapes effort, persistence, and action choice. Within academics, self-efficacy consistently links to enhanced engagement, empowering students to embrace challenging tasks and persevere (Pajares, 1996; Pintrich and De Groot, 1990; Schunk and Zimmerman, 1998). Our observed mediating effect aligns with existing research positioning self-efficacy as an intermediary between motivational constructs and academic outcomes (Li, 2023; Yau and Shu, 2023). For example, prior studies suggest enjoyment or reduced boredom mediate growth mindset's link to engagement (Derakhshan et al., 2024; Li, 2023), while negotiable fate and self-efficacy mediate grit's influence (Derakhshan and Fathi, 2024; Yau and Shu, 2023). Our finding that self-efficacy partially mediates both relationships is a noteworthy addition, suggesting its significant, though not exclusive, mechanistic role. The partial mediation, evidenced by continued direct effects of growth mindset and grit on engagement, indicates other operative pathways. This aligns with literature acknowledging that motivational constructs often exert both direct and indirect influences (Meng and Zhang, 2023). For instance, a growth mindset might directly foster inherent love for learning, while grit could sustain engagement through sheer determination, irrespective of self-efficacy levels. EMA entries provided compelling direct evidence of this mediation, illustrating how a sudden boost in confidence after solving a problem immediately propelled students into deeper, more sustained engagement, and conversely, how a dip in self-efficacy led to disengagement in real-time.

To further illuminate these quantitative findings, our qualitative data from focus groups and EMA diaries provided rich contextual nuances regarding how these constructs manifest in students' real-world academic experiences. Three overarching themes emerged from thematic analysis, reinforcing and expanding upon the quantitative results. The first, “Embracing Challenges and Setbacks as Learning Opportunities,” captured how growth mindset and grit manifest. Students articulated perceiving academic challenges as valuable avenues for personal and intellectual growth, demonstrating resilience. These narratives strongly echo Dweck's (2006) emphasis on effort and learning from failure, and Duckworth et al. (2007) definition of grit as unwavering perseverance. Students recounted diligently working through demanding coursework, driven by belief in their capacity to improve, illustrating a clear synergy. This qualitative evidence humanizes statistical relationships, providing concrete examples of these traits in authentic academic settings. However, a subtle nuance emerged: some students, like Fang, expressed concern about potential excessive pressure and burnout from relentless self-improvement in highly competitive environments. This highlights the need for a balanced perspective in applying these constructs, especially under high academic pressure. EMA entries consistently demonstrated these rapid, *in-situ* cognitive reframings of challenges into learning opportunities, providing immediate “snapshots” of growth mindset and grit in action within daily academic tasks, often revealing how minor daily setbacks were met with immediate strategies of perseverance.

The second prominent theme, “Self-Efficacy as the Cornerstone of Proactive Engagement,” underscored academic self-efficacy's vital role in driving active student involvement. Students consistently described how confidence in their academic skills motivated proactive engagement, encouraging class participation, challenging opportunities, and persistence. This directly supports Bandura's (1997) assertion regarding self-efficacy's influence on behavior initiation and maintenance, aligning with our quantitative finding of its strong positive effect on engagement. For example, a student recounted feeling empowered to participate in group projects after mastering a challenging concept, vividly illustrating how self-efficacy translates belief into tangible academic actions. This qualitative theme provides a valuable lens for understanding why self-efficacy functions as a critical link between motivational beliefs and observable academic engagement. The EMA data provided particularly strong evidence for this, showcasing the direct, immediate impact of perceived competence on behavioral choices, where moments of self-efficacy directly correlated with opting for more difficult tasks or extending study sessions, while moments of low self-efficacy were immediately followed by disengagement or procrastination.

Finally, the theme of “Cultural and Contextual Influences on Motivational Beliefs and Engagement” brought to the forefront the significant impact of the Chinese educational context. Participants frequently referenced deeply ingrained cultural values—such as collectivism, filial piety, and the pronounced societal emphasis on academic achievement—as key factors shaping their approaches to learning and motivation. Many students highlighted achieving high grades as a way to honor their families, reflecting a strong collectivist orientation that amplified their intrinsic academic motivation. This finding aligns with scholarly calls for culturally sensitive approaches to student motivation (Santos et al., 2023), suggesting that while core principles of growth mindset, grit, and self-efficacy hold broad relevance, their specific expression and impact are demonstrably shaped by cultural factors. In China's intensely competitive academic environment, coupled with strong familial expectations, these motivational constructs' effects may be particularly pronounced, offering a unique cultural vantage point for interpreting their influence on student engagement. While EMA prompts did not explicitly ask about cultural values, the recurring, spontaneous mentions of familial expectations and societal contributions within daily reflections, often in conjunction with motivation to overcome challenges, subtly but powerfully reinforced the pervasive influence of these cultural factors in real-time academic experiences.

Beyond reinforcing the focus group insights, the EMA component offered unique contributions by illuminating the intra-individual variability in motivation and engagement throughout a typical day or week. It revealed the micro-processes by which growth mindset, grit, and self-efficacy are activated or suppressed in specific academic moments, providing a dynamic “process-level” understanding that static surveys and retrospective interviews cannot fully capture. This granular data revealed the fluidity of these constructs and the immediate cognitive and emotional responses that mediate their impact on engagement, offering a richer, more ecologically valid perspective on student learning experiences.

In summary, the qualitative findings from the focus group interviews and EMA diaries richly complement and extend the quantitative results. The thematic analysis provides compelling first-person accounts illustrating how growth mindset and academic grit manifest in students' approaches to challenges, how self-efficacy mediates these beliefs into active academic engagement, and how cultural context shapes the motivational landscape. The EMA component further illuminates the dynamic, real-time activation of these constructs and their direct impact on daily engagement. The qualitative data confirms the positive relationships identified in the SEM analysis, offering nuanced insights into the lived experiences and contextual factors underpinning these relationships, thereby providing a more holistic and ecologically valid understanding of student motivation and engagement in mainland Chinese universities.

## 6 Implications

Our findings call for a systemic shift in educational practices to create learning environments that intrinsically cultivate student motivation and engagement. Moving beyond isolated interventions, the results have significant implications for pedagogical design, assessment methods, and institutional support within the Chinese higher education context.

Rather than adding standalone workshops, educators can embed these principles into the very fabric of their courses. This involves designing a “growth-oriented curriculum” where assignments are iterative, allowing for mistakes, feedback, and revision, which normalizes challenges as part of the learning process. For instance, interventions providing “wise feedback”—which frames critical feedback as a sign of the instructor's high standards and belief in the student's potential to meet them—have been shown to boost motivation and achievement (Yeager et al., 2014). The crucial mediating role of self-efficacy also calls for structuring courses around “mastery-oriented learning pathways.” By carefully scaffolding content to ensure early successes, educators can build student confidence and create a positive motivational cycle that aligns with established principles of self-regulated learning (Zimmerman and Schunk, 2001). Furthermore, implementing long-term, problem-based learning can inherently foster academic grit, as it requires sustained effort to solve complex, authentic problems, a process known to develop persistence in students (Dochy et al., 2003).

Our results challenge the over-reliance on traditional summative exams and suggest a move toward more process-oriented and formative assessment. If mindset and grit are valued, then assessment criteria should be expanded to formally recognize and reward elements like effort, improvement over time, and thoughtful revision—not just the final correct answer. This “assessment for learning” approach uses feedback to build student capacity rather than simply measure it (Boud and Molloy, 2013). The EMA findings, which highlighted the immediate impact of small successes and failures on motivation, provide a powerful rationale for implementing more frequent, low-stakes assessments. This approach provides the timely, specific feedback necessary to manage real-time fluctuations in self-efficacy and sustain daily engagement (Nicol and Macfarlane-Dick, 2006).

Finally, the impact of these findings extends beyond the classroom to institutional policy. The powerful influence of cultural values like filial piety suggests that universities should create structured mentorship programs that connect students with alumni who model both professional success and social responsibility. This helps students frame their academic goals within a larger purpose, a practice shown to enhance student retention and sense of belonging (Crisp et al., 2017). Moreover, our qualitative data hinted at the risk of student burnout from a relentless focus on effort. This indicates that university support services should offer culturally-tailored workshops on resilience and managing academic pressure, creating a holistic ecosystem that not only promotes motivation but also protects student wellbeing, for which positive psychology interventions have shown considerable promise in university settings (Hobbs et al., 2022).

## 7 Limitations and future directions

This study, despite its contributions, has limitations that inform future research. First, our cross-sectional design prevents causal inferences. Future longitudinal studies are crucial to understand the temporal dynamics of growth mindset, grit, self-efficacy, and academic engagement in Chinese undergraduates. Tracking students through academic transitions, and conducting longer-term EMA studies, would offer clearer insights into these constructs' persistence and fluctuations within the demanding Chinese higher education system.

Second, the sample, while geographically diverse within mainland China, was limited to three universities. This restricts generalizability even within China, given vast regional differences. Future research should include a broader range of institutions (e.g., diverse tiers, eastern vs. western regions) and engage in cross-cultural comparative studies. Comparing Chinese undergraduates with students from other cultural contexts could illuminate how cultural differences moderate the effects of these constructs and refine our understanding of their culturally nuanced expressions. Third, the qualitative phase, though insightful, involved a small number of focus groups and a 2-week EMA period for a subset. Future studies could employ more extensive qualitative methods. Individual semi-structured interviews with a larger, more diverse sample could reveal richer, individualized narratives. Ethnographic observations of classroom interactions would provide valuable “ground-level” perspectives, triangulating data with observed behaviors. While EMA offers real-time insights, its reliance on self-report suggests future studies could integrate objective measures of engagement (e.g., task completion logs, learning platform analytics) for multi-modal assessment.

A significant limitation is the focus on self-efficacy as the sole mediator. Our partial mediation findings suggest other mechanisms are likely involved. Given China's cultural emphasis, future research should explore the mediating roles of factors like intrinsic motivation, achievement goal orientations (with cultural nuances), and perceived social support from family and peers. Developing and testing more comprehensive mediation models incorporating these pathways would enrich our understanding. Examining how classroom climate, teacher-student relationships, or peer influences interact with growth mindset and grit could also reveal crucial contextual moderators. EMA could further investigate how momentary positive affect or coping strategies mediate real-time links between challenges and engagement.

Finally, broader methodological discussions, like those concerning growth mindset interventions' efficacy (Macnamara and Burgoyne, 2023), underscore the need for rigor. Future research in the Chinese context should prioritize experimental designs, such as randomized controlled trials of interventions, to establish causality and address biases. Additionally, exploring “mixed mindsets” (Chaffee and Noels, 2022)—the co-existence of growth and fixed beliefs—in Chinese undergraduates, and how cultural values interact with these nuanced profiles, would offer a more refined, culturally sensitive understanding for developing effective interventions.

## Data Availability

The data analyzed in this study is subject to the following licenses/restrictions: The datasets used and/or analyzed during the current study are available from the corresponding author on reasonable request. Requests to access these datasets should be directed to Haizhen Liang, janejane0414@163.com.

## Appendix

### Appendix A: Focus Group Interview Protocol

**Moderator's Introduction Script:** “Hello everyone, thank you for joining us today. My name is [Moderator's Name], and we're here to talk about your experiences as university students—specifically your thoughts on learning, motivation, and challenges. There are no right or wrong answers; we are simply interested in your honest perspectives. This discussion is confidential, and your names will not be used in our report. Please feel free to speak openly and also listen to the experiences of others. Does anyone have any questions before we begin?” **Interview Questions (with Mandarin Chinese Translation) Part 1: Warm-up & General Academic Experience**

1. To begin, could you briefly describe what your academic experience at university has been like so far? (首先,请大家简单描述一下你们到目前为止的大学学习体验是怎样的 ?)
2. What do you enjoy most about your studies, and what do you find most challenging? (在学习中,你们最享受的是什么？觉得最具挑战性的又是什么？) **Part 2: Exploring Growth Mindset & Academic Grit**
3. When you face a very difficult topic or assignment in your major, what typically goes through your mind? What's your first reaction? (当你们在专业课中遇到一个非常难的课题或作业时, 通常会想些什么？你们的第一反应是什么？)
4. Think about your own abilities in your field of study. Do you see them as something that is relatively fixed, or as something you can significantly develop over time? Can you share an example that illustrates your view? (思考一下你们在自己专业领域的能力。你们觉得这些能力是相对固定的,还是可以通过 时间显著发展的？可以分享一个例子来说明你的看法吗？)
5. Can you tell us about a time you experienced an academic setback or failure, like a poor grade on an important exam? How did you handle it? (可以分享一次你经历学业挫折或失败的经历吗？比如一次重要考试成绩不理想。你当时 是如何应对的？)
6. Persistence, or “grit,” is often talked about. Can you describe a situation where you had to persevere for a long time on a difficult project or course? What kept you going? (我们常说的“毅力”或“坚韧”。你能描述一个你曾为了一个困难项目或课程而长期坚持的 经历吗？是什么让你坚持下来的？) **Part 3: Exploring Academic Self-Efficacy & Engagement**
7. How does your level of confidence in a subject influence how you behave in that class? For example, does it affect whether you ask questions or participate in discussions? 你们在某一科目上的自信程度是如何影响你们在该课堂上的行为的？例如,这会影响你们提问或参与讨论的积极性吗？)
8. Think of a time when feeling very confident in your abilities helped you become more engaged or interested in a topic. Can you describe that experience? (回想一次经历,那次你对自己的能力充满信心,这种自信帮助你更深入地投入或对一个课题更感兴趣。可以描述一下那次经历吗？) **Part 4: Exploring Cultural & Contextual Factors**
9. Besides your own personal career goals, what are some of the other important motivators for you to succeed at university? (除了你自己的职业目标之外,还有哪些其他重要的动力激励你在大学里取得成功？)
10. How do the expectations of others, like your family, teachers, or even society in general, play a role in your academic life and motivation? (在你看来,来自他人(如家庭、老师, 乃至整个社会)的期望在你的学习生活和动机中扮演着什么样的角色？) **Part 5: Closing**
11. Is there anything else you'd like to share about student motivation, challenges, or engagement that we haven't talked about today? (关于学生动机、挑战或学习投入,今天我们没有讨论到的,还有没有什么你们想分享的?)
